# Physiological responses and antioxidant properties of coriander plants (*Coriandrum sativum* L.) under different light intensities of red and blue lights

**DOI:** 10.1038/s41598-022-25749-3

**Published:** 2022-12-07

**Authors:** Hsin-Hung Lin, Kuan-Hung Lin, Meei-Ju Yang, Hoang Chinh Nguyen, Huei-Ju Wang, Han-Xuang Huang, Meng-Yuan Huang

**Affiliations:** 1 Department of Agronomy, National Chung Hsing University, Taichung, 40227 Taiwan; 2 Department of Horticulture and Biotechnology, Chinese Culture University, Taipei, 11114 Taiwan; 3 Tea Research and Extension Station, Yangmei, Taoyüan City, 326011 Taiwan; 4 School of Life and Environmental Sciences, Deakin University, Geelong, VIC 3216 Australia; 5 Department of Applied Science of Living, Chinese Culture University, Taipei, 11114 Taiwan; 6 Department of Life Sciences and Innovation and Development Center of Sustainable Agriculture, National Chung Hsing University, Taichung, 40227 Taiwan

**Keywords:** Physiology, Plant sciences

## Abstract

Coriander (*Coriandrum sativum* L.) contains abundant antioxidants and essential oils which can provide antibacterial, antifungal, and antioxidant activities in the pharmaceutical, health and food production industry. To improve the economic values of coriander, the relationships between optimal light treatments for maximizing both plant growth and the antioxidant and essential oil content of coriander leaves need to be determined. Plants were exposed to five light-emitting diodes spectral color mixtures, high blue light (BL) intensity induced the levels of reducing power response. The light treatments were then adjusted for the analysis of secondary metabolite compounds of coriander leaves. Among 30 identified compounds, the amounts of decamethyl-cyclopentasiloxane and dodecane were significantly reduced in the R80 + G50 + B50 condition, whereas dodecamethyl-cyclohexasiloxane level was significantly reduced in R50 + G50 + B80 condition. Various light quality and intensity combinations influenced the accumulations of chlorophyll and phytochemical contents, mediated antioxidative properties, and secondary metabolites of coriander leaves, which may be useful in developing a new LED lighting apparatus optimized for coriander production in plant factories.

## Introduction

Coriander (*Coriandrum sativum* L.) is an aromatic and herbaceous annual crop in the family of Apiaceae. The fresh leaves and spice seeds of coriander are standard ingredients in many of the curry powders^1^. In addition, coriander plants are well-known for their medicinal, perfume, cosmetic, and secondary metabolic compounds, such as volatile components, flavonoids, linolenic acid, β-carotene, and phenolic compounds^2^. The essential oil and extracts from coriander plants provide promising antibacterial, antifungal, and antioxidant activities^3^. They are not only used to keep food from spoilage, but also treated as pharmaceutical uses, alternative medicine, and natural therapies treatments to help human against pathogen infections^4^. There is an increasing interest in natural food additives, which can function as natural antioxidants, in addition to seasoning the foods. Various environmental factors not only influence the accumulation of photosynthetic pigments and phytochemicals^5^, but also mediate antioxidant capacities in plant leaves. Among them, the light environment is one of the most significant factors influencing changes in plant growth, secondary metabolite accumulation, and physiological occurring in plants as they are exposed to varying light quality and intensity^6^. Thus, the optimization of light treatment is the first urgent task in maximizing the economic output of plant production and quality.

Lights, especially blue light (BL) and red light (RL), are important environmental signals for various organisms of plants because they regulate plant developmental and physiologic processes though photoreceptors^7^, and play major roles in the action spectra for energy sources of photosynthetic CO_2_ assimilation that can provide great impacts on plant growth^8^. Light-emitting diodes (LED) are semiconductor device which can emit light with various wavelengths, and perform better energy efficiency and lower cost than high-pressure sodium lamps. LED technology provides possible solutions for more sustainable greenhouse production not only it reduces carbon footprint of the individual light photon but plants are highly responsive to changes in light quality and intensity. The output light intensity and wavelengths from LED device can be selected to maximize the crop yield and the accumulation of antioxidants and overcome the stress conditions as well. Verma et al.^9^ indicated that the mixture of RL and BL (4:1) LED lights significantly increased the leaf number, root length, leaf width, stomata width, leaf area, and the fresh and dry weights of leaf and root of foxglove (*Digitalis purpurea*) compared to RL or BL alone, and fluorescent lamp treatments. The quercetin 3-rutinoside (rutin) is a tomato natural flavonoid, and the applications of rutin can increase plant growth, photosynthesis, and fruit quality^10^. Groher et al.^11^ also reported that the concentrations of rutin in tomato leaves was increased by RL:BL (4:1) LED when grown in the greenhouses. Loughrin and Kasperbauer ^12^ revealed that the basil (*Ocimum basilicum*) leaf size, aroma, and the soluble phenolics and antioxidants concentrations were affected by light quality. Taulavuori et al.^13^ showed that the production of basil phenolic acids was increased when plants were treated with extra BL which supplied with traditional high-pressure sodium lamps. Bantis et al.^14^ also illustrated that the growth characteristics and total phenolic content of basil cultivars were affected by different LED light treatments. Naznin et al.^15^ stated that the coriander (*Coriandrum sativum* L.) plant could yield the highest biomass when exposed to RL: BL (10:1) LED, and could accumulate highest antioxidant activity when exposed to RL:BL (5:1) LED. In addition, the growth of coriander plants was affected by interaction between root-zone temperature (RZT) and photosynthetic photon flux density (PPFD), and that the combination of RZT at 25 °C and PPFD at 300 μmol m^−2^ s^−1^ gave the greatest plant growth, but total phenolic concentrations and the antioxidant capacity of the coriander plant were greatest under the combination of 300 μmol m^−2^ s^−1^ and 30 °C compared to control^16^. Bian et al.^17^ reported that the spectra of green light (GL) could induce different responses of morphogenesis and photosynthesis in lettuce (*Lactuca sativa* L.). The GL not only to enhanced the photosynthetic capacity including increasing the efficiency of net photosynthetic rates, maximal photochemical efficiency, and electron transport for carbon fixation and chlorophylls (Chl) content, but are also reduced the accumulation of malondialdehyde and H_2_O_2_ contents though increasing the superoxide dismutase (SOD) and ascorbate peroxidase (APX) activities. Moreover, Lanoue et al.^18^ suggested that when designing lighting systems for optimizing leaf strength during plant production, the addition of GL LED to the traditionally used RL and BL LED should be considered and tested in environmentally controlled systems.

Recently, we illustrated that a RL:BL:GL LED ratio of 4:1:1 treatment resulted in many positive effects on the growth, development, and appearance of basil plants (*Ocimum basilicum*) compared to 2:1:1 or 1:1:1 treatment^6^. Light treatments significantly affected pigments and cultivars differently, in that purple and green basils displayed remarkably higher and lower pigment content under 2:1:1 and 1:1:1 treatment, respectively. In addition, we also reported that 40 PPFD of the BL caused greater accumulation of dry biomass and soluble sugars of the edible oyster mushrooms (*Lentinus sajor-caju*) than other BL intensities^19^. Moreover, we found that the soluble sugar and nitrate contents in the lettuce plants (*Lactuca sativa* L.) grown under white broad-spectrum light (WL) supplied to an RL plus BL LED treatment were significantly higher and lower, respectively, compared to those under RL plus BL treatment. However, the Chl, carotenoids (Car), and soluble protein contents of lettuce leaves showed no significant differences among these treatments^5^. Although light spectra have been used for studying the productivity and antioxidant properties of these plants, different mixtures of RL and BL LED under various light intensities for growing coriander plants remain unknown. In this study, we chose different proportions of RL and BL irradiation and supplement it with GL under different light intensities to identify the optimal light quality conditions for growing coriander to maximize production and antioxidant accumulation. The hypothesis of this study was that physiological response and antioxidant capacity of corianders would differ under RL, BL and GL LED of various intensities. The ultimate goal of the research was to develop a year-round production system for fresh, highly nutritious, economically feasible coriander plants produced in close proximity to the final retail market. An optimal strategy for regulating light quality and intensity will help in designing growth chambers and greenhouse light environments to obtain maximum economic benefits for coriander growers.

## Results

### Plant growth and morphological performances

**Figure 1:**
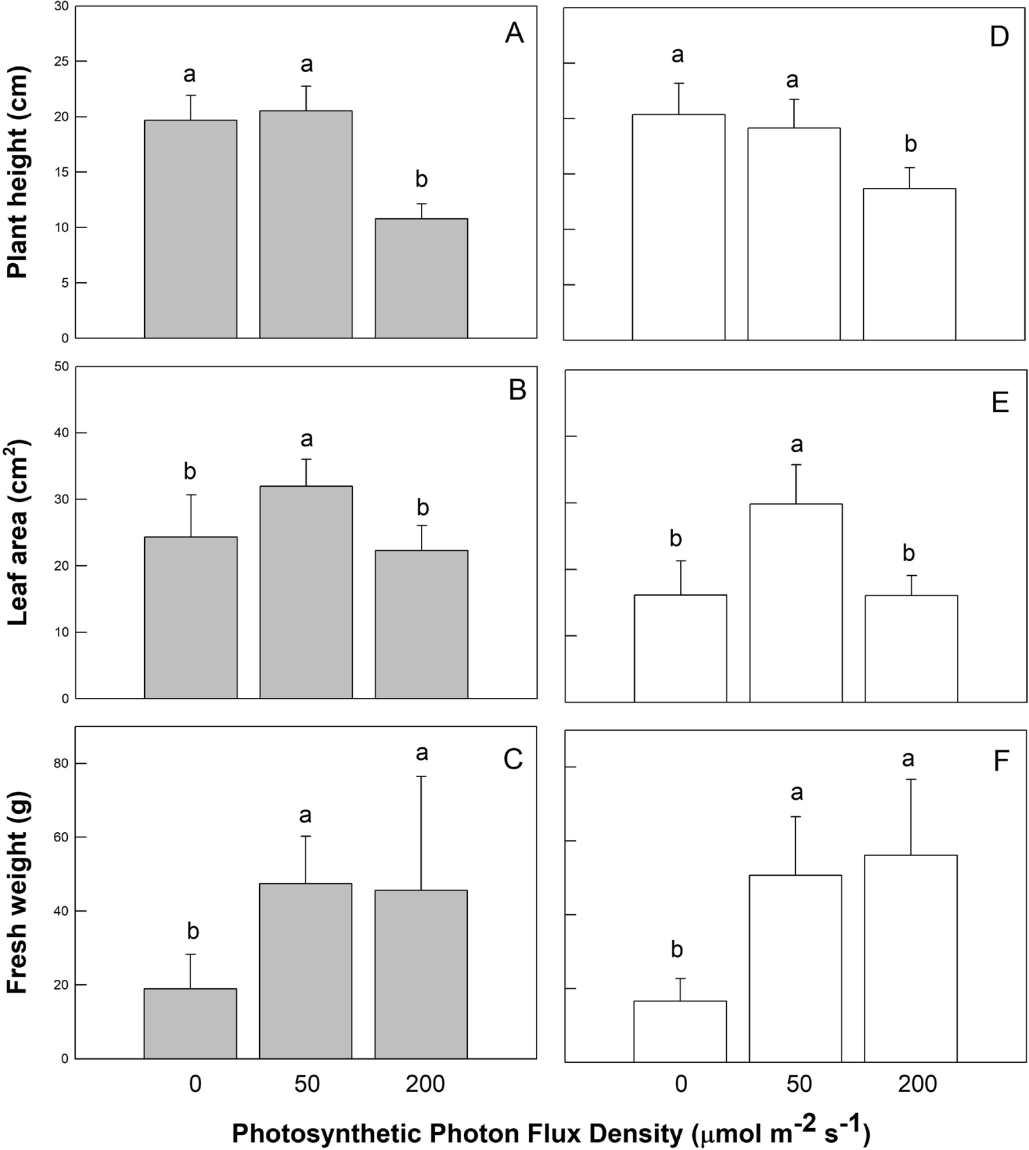
Effects of LED light quality (RL, GL, and BL) and intensity (0, 50, and 200 µmol m^−2^ s^−1^ photosynthetic photon flux density) treatments on plant height (panels **A**, **D**), leaf area (panels **B**, **E**), and shoot fresh weight (panels **C**, **F**) of coriander plants at 45 days after sowing. There are five light quality treatments consisting of red light (RL), green light (GL), and blue light (BL) LED with different mixtures. The light treatments associated with the plant growth measurements presented from left to right were no RL (G50 + B50), control (R50 + G50 + B50), high RL (R200 + G50 + B50), no BL (R50 + G50), control (R50 + G50 + B50), and high BL (R50 + G50 + B200) treatments, respectively. Treatments were arranged in a completely randomized design with 3 replicates, and 10 plants from each light quality and intensity treatment were used for the plant growth measurements. Vertical bars indicate the standard deviation. The values followed by a different letter show statistically significant differences at *p* < 0.05 (n = 10).

Coriander RV-164 plant is a popular variety grown in Taiwan for consumption as fresh vegetables. To study the effects of light treatments of coriander plants were monitored by measurements of changes in the growth parameters (plant height, LA, and shoot FW), showing distinct growth responses to different light quality and light intensity treatments (Fig. 1). All plant height parameters of coriander grown under high RL and high BL lightings under 200 μmol m^−2^ s^−1^ PPFD had significantly smaller values (10.8 ± 1.37 and 13.7 ± 1.90 cm, respectively) than those under other light treatments (19.7 ± 2.24 ~ 20.5 ± 2.25 cm). Furthermore, LA values of all coriander plants were significantly larger when grown under control conditions (31.9 ± 4.03 and 29.8 ± 5.88 cm^2^) compared to other treatments (16.0 ± 5.13 – 24.3 ± 6.36 cm^2^). The shoot FW of all coriander plants was significantly lower in no RL and no BL lightings (18.9 ± 9.28 and 16.5 ± 6.22 g, respectively) than in other treatments (45.6 ± 30.57 ~ 56.1 ± 20.49 g), indicating that shoot FW in coriander was reduced in the absence of RL and BL treatments.

### Chl and phytochemical contents of coriander plant

**Figure 2:**
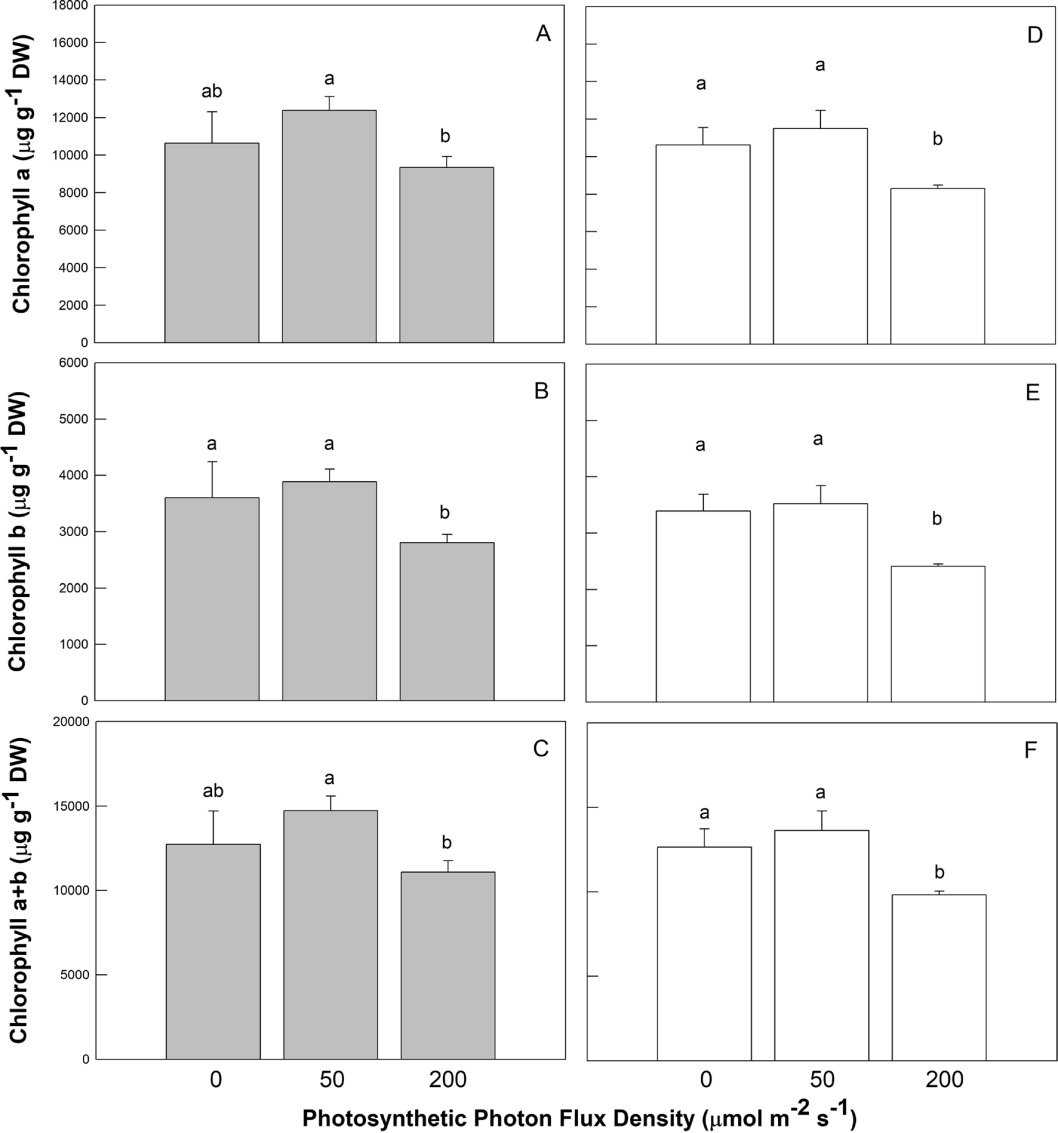
Effects of LED light quality (RL, GL, and BL) and intensity (0, 50, and 200 µmol m^−2^ s^−1^ photosynthetic photon flux density) treatments on chlorophyll a (panels **A**, **D**), chlorophyll b (panels **B**, **E**), and chlorophyll a + b (panels **C**, **F**) of coriander plants at 45 days after sowing. There are five light quality treatments consisting of red light (RL), green light (GL), and blue light (BL) LED with different mixtures. The light treatments associated with the plant chlorophyll measurements presented from left to right were no RL (G50 + B50), control (R50 + G50 + B50), high RL (R200 + G50 + B50), no BL (R50 + G50), control (R50 + G50 + B50), and high BL (R50 + G50 + B200) treatments, respectively. Treatments were arranged in a completely randomized design with 3 replicates, and 10 plants from each light quality and intensity treatment were used for the plant chlorophyll measurements. Vertical bars indicate the standard deviation. The values followed by a different letter show statistically significant differences at *p* < 0.05 (n = 10).

**Figure 3:**
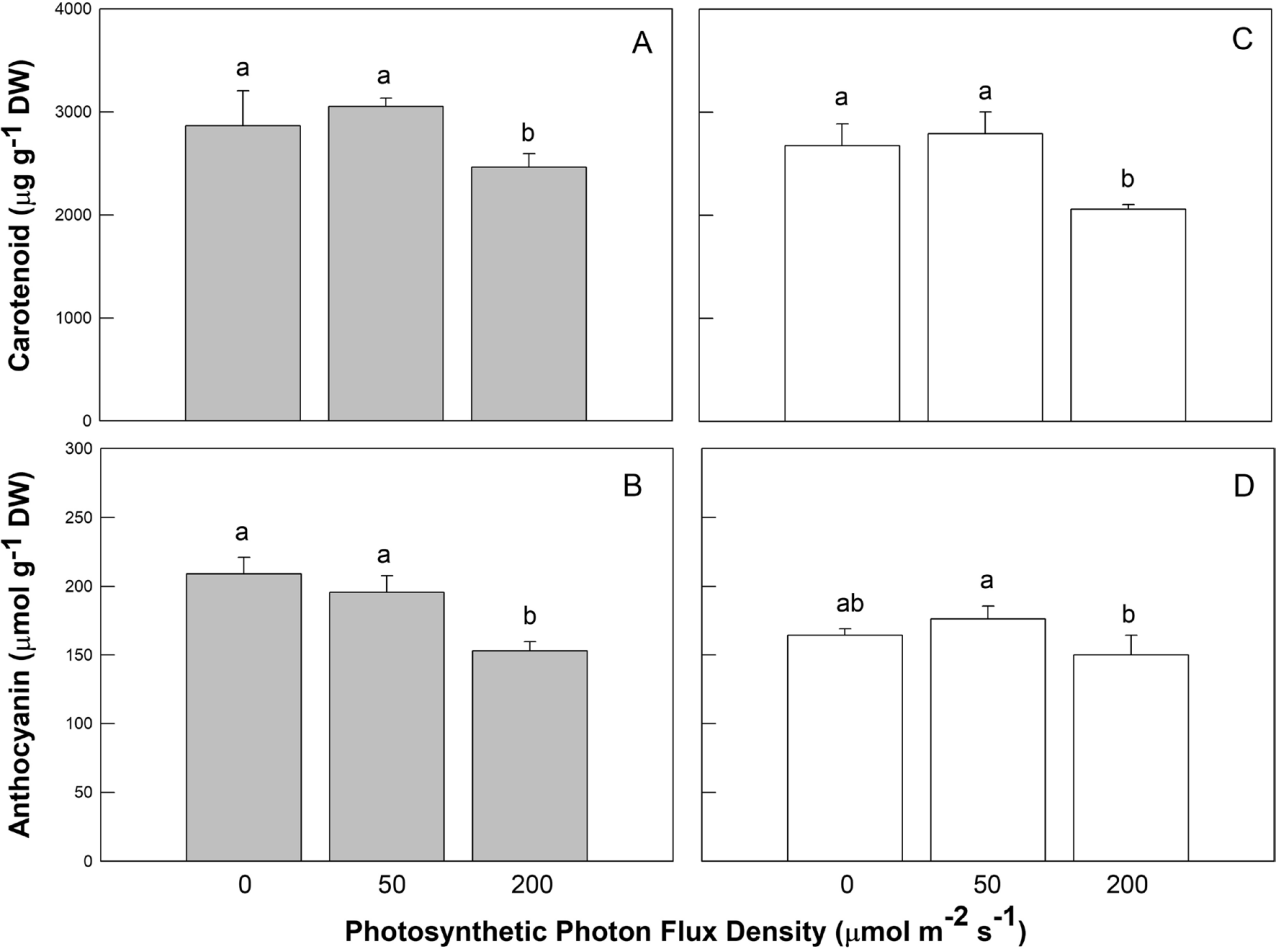
Effects of LED light quality (RL, GL, and BL) and intensity (0, 50, and 200 µmol m^−2^ s^−1^ photosynthetic photon flux density) treatments on carotenoid (panels **A**, **C**), and anthocyanin (panels **B**, **D**) of coriander plants at 45 days after sowing. There are five light quality treatments consisting of red light (RL), green light (GL), and blue light (BL) LED with different mixtures. The light treatments associated with the plant phytochemical measurements presented from left to right were no RL (G50 + B50), control (R50 + G50 + B50), high RL (R200 + G50 + B50), no BL (R50 + G50), control (R50 + G50 + B50), and high BL (R50 + G50 + B200) treatments, respectively. Treatments were arranged in a completely randomized design with 3 replicates, and 10 plants from each light quality and intensity treatment were used for the plant phytochemical measurements. Vertical bars indicate the standard deviation. The values followed by a different letter show statistically significant differences at *p* < 0.05 (n = 10).

**Figure 4:**
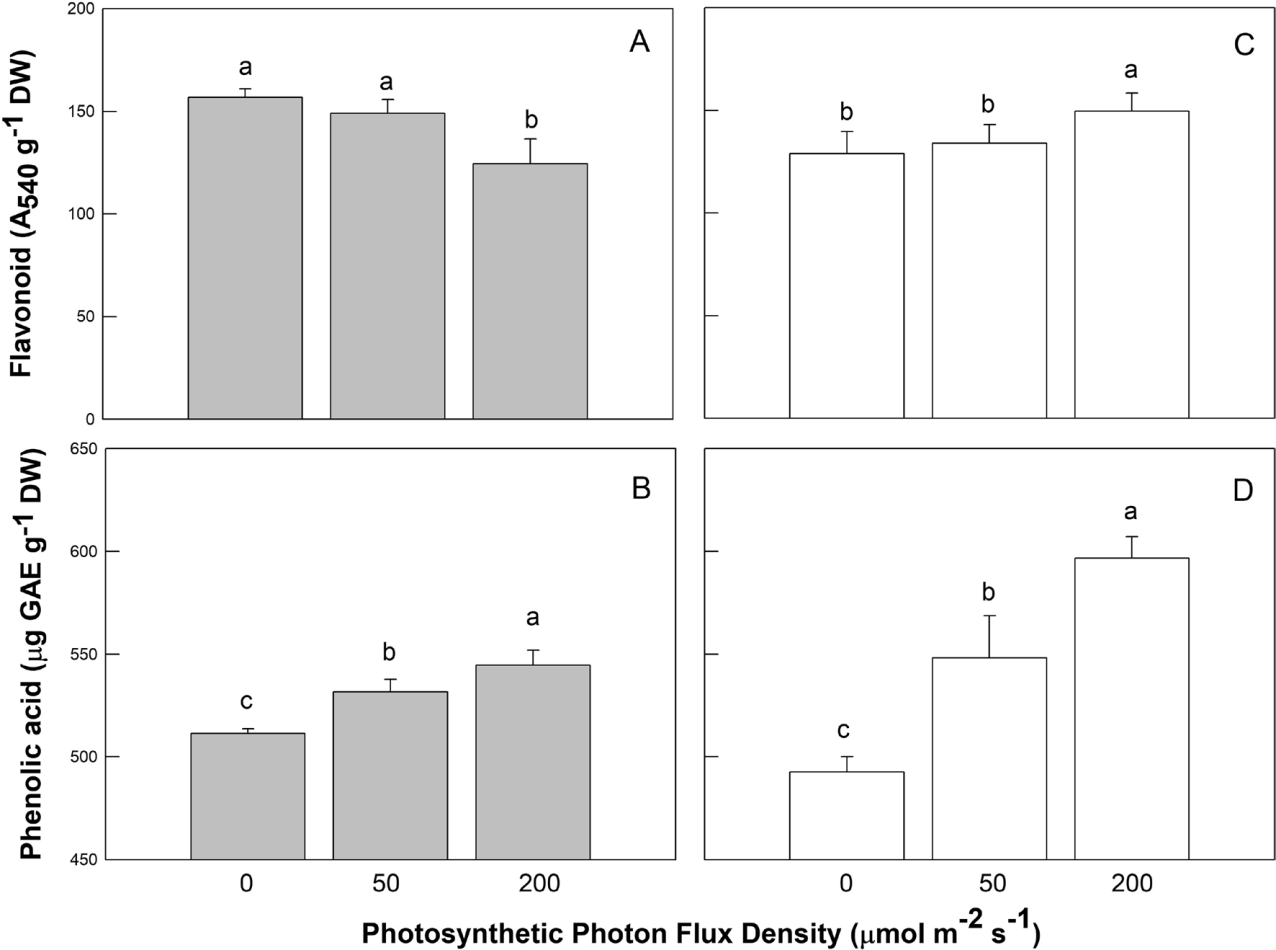
Effects of LED light quality (RL, GL, and BL) and intensity (0, 50, and 200 µmol m^−2^ s^−1^ photosynthetic photon flux density) treatments on flavonoid (panels **A**, **C**), and phenolic acid (panels **B**, **D**) of coriander plants at 45 days after sowing. There are five light quality treatments consisting of red light (RL), green light (GL), and blue light (BL) LED with different mixtures. Light treatments associated with the antioxidant measurements presented from left to right were no RL (G50 + B50), control (R50 + G50 + B50), high RL (R200 + G50 + B50), no BL (R50 + G50), control (R50 + G50 + B50), and high BL (R50 + G50 + B200) treatments, respectively. Treatments were arranged in a completely randomized design with 3 replicates, and 10 plants from each light quality and intensity treatment were used for the plant antioxidant measurements. Vertical bars indicate the standard deviation. The values followed by a different letter show statistically significant differences at *p* < 0.05 (n = 10).

The Chl and phytochemical content of coriander plants cultured under mixtures of RL, BL and GL LED of various light intensities are shown in Figs. 2, 3, and 4. The leaf Chl a, b, and a + b contents in plants grown under control conditions were highest (Fig. 2), in comparison with other treatments. Chl a, b, and a + b values observed under control conditions were 12,378 ± 732 (Fig. 2A), 3,885 ± 221 (Fig. 2B), and 14,718 ± 868 (Fig. 2C) μg g^−1^ DW, respectively. The same trend was also observed in the BL treatment group (Fig. 2D–F). Significantly lower levels of all Chl contents in leaves were found when plants were grown under high light intensity compared to the control and low light intensity treatment. However, no significant differences were observed in all Chl contents among control, no RL (Fig. 2A–C, and no BL (Fig. 2D–F) of light spectra under low light intensity.

Figure 3 presents Car and Ant content of coriander plant leaf extracts. All phytochemical pigment contents in high PPFD treatment with high RL (Fig. 3A, B) and high BL (Fig. 3C, D) were significantly lower than in control and other light treatments, with the exception that no significant differences were observed in Ant contents between no BL by low PPFD (164.4 ± 4.7 μmol g^−1^ DW) and high BL by high PPFD (150.1 ± 10.4 μmol g^−1^ DW) treatments (Fig. 3D).

Figure 4 demonstrates that coriander plants under high PPFD with BL contained significantly higher levels of flavonoids (Fla) (Fig. 4C) and phenolic acid (Pha) (Fig. 4D) compared to controls and other treatments, whereas Fla levels under high PPFD with RL were significantly lower than that under control and no RL (Fig. 4A) and phenolic acid (Pha) content was higher for plants under high RL conditions (Fig. 4B).

### Antioxidant properties of coriander plant

**Figure 5:**
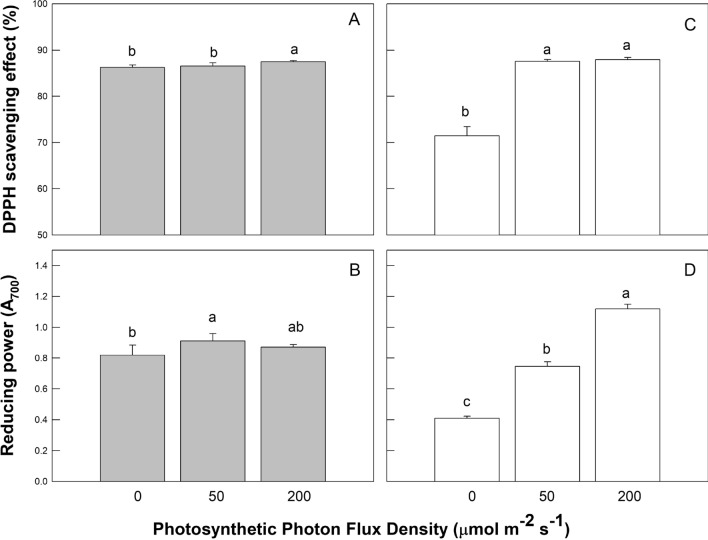
Effects of LED light quality (RL, GL, and BL) and intensity (0, 50, and 200 µmol m^−2^ s^−1^ photosynthetic photon flux density) treatments on DPPH scavenging effect (panels **A**, **C**), and reducing power (panels **B**, **D**) of coriander plants at 45 days after sowing. There are five light quality treatments consisting of red light (RL), green light (GL), and blue light (BL) LED with different mixtures. The light treatments associated with the plant antioxidant capacity measurements presented from left to right were no RL (G50 + B50), control (R50 + G50 + B50), high RL (R200 + G50 + B50), no BL (R50 + G50), control (R50 + G50 + B50), and high BL (R50 + G50 + B200) treatments, respectively. Treatments were arranged in a completely randomized design with 3 replicates, and 10 plants from each light quality and intensity treatment were used for the plant antioxidant capacity measurements. Vertical bars indicate the standard deviation. The values followed by a different letter show statistically significant differences at *p* < 0.05 (n = 10).

CorianderS are herbaceous plants providing nutraceutical products with antioxidant properties. The DPPH free radical scavenging assay was used to determine the antioxidant activity of plant extracts. Significantly higher DPPH scavenging effect was observed in those plants cultured under high RL (87.5 ± 0.3%) and high BL (87.9 ± 0.3%) at high PPFD than in plants receiving no RL (86.2 ± 0.5%) and no BL (71.5 ± 1.9%) at low PPFD (Fig. 5A, C). However, differences in the DPPH scavenging effect observed between the no RL treatment and the control (Fig. 5A) and between the control and the high BL treatment (Fig. 5C) were not significant. The levels of reducing power in coriander leaves were significantly higher in the controls than in the no RL and no BL treatments at low PPFD (Fig. 5B, D). In addition, the reduction power was about 2.73 times higher in plants under high BL at high PPFD than in the plants under no RL at low PPFD (Fig. 5D).

### Chemical composition of secondary metabolite accumulation in coriander leaf

A total of 30 compounds were detected by GC–MS. Only the compounds which appeared in each of the 5 freeze-dried replicated plant samples processed, and the average peak area (absorbance) of identified compound accumulation performed for 160 min are shown in Table 1. A total of 15 compounds were also identified that occurred in only one or two of the 5 plant samples, therefore, it is not possible to calculate the significant differences between light treatments for these compounds (Table S1). Notably, β-cyclocitral (RT 48.946 min) was not detected in the R20 + G50 + B50, R50 + G50 + B20 light intensity and control (Table S1). In Table 1, among those 15 identified compounds, a large effect of decamethyl-cyclopentasiloxane (DCP), dodecane (DO), and dodecamethyl-cyclohexasiloxane (DOCP) content in coriander leaves was observed under various light treatments. The total peak area of DCP, DO, and DOCP isolated was identified after 45.36, 47.913, and 58.193 min extraction times, respectively. The area of DCP was significantly (*p* < 0.05) lower for plants receiving R80 + G50 + B50 light intensity (0.53 ± 0.103%) than for plants receiving R20 + G50 + B50 light intensity (0.764 ± 0.229%), whereas the area of DOCP was significantly (*p* < 0.05) lower under R50 + G50 + B80 light intensity (0.276 ± 0.088%) than under R50 + G50 + B20 light intensity (0.551 ± 0.168%). Moreover, both DCP and DOCP decreased with increasing in R light and B light intensity, respectively. However, significantly (*p* < 0.001) higher DO area was observed in the control (0.513 ± 0.128%) compared to other light treatments. In addition, when plants receiving R20 + G50 + B50 and R80 + G50 + B50 light intensity, the area of DCP was 28% more and 30% lower than control (receiving R50 + G50 + B50 light intensity). Nevertheless, when plants receiving R50 + G50 + B20 and R50 + G50 + B80 light intensity, the area of DOCP was 33% more and 26% lower than for control. The areas of the rest of the detected compounds in coriander leaves did not show any significant differences among treatments, suggesting that light treatments did not influence the amounts of these 12 identified compounds, although some of them (i.e., decanal, (E)-2-decenal, nonane, and 1-decanol) showed high absorbance area (> 2 × 10^8^) in most of light treatments.

**Table 1:** The chemical composition of coriander leaf essential oil extracted from freeze-dried samples of 5 plants under different light treatments.

RT (min)	CAS number	Compound	Relative percentage (%)			Relative percentage (%)		
			Low RL R20 + G50 + B50	Control R50 + G50 + B50	High RL R80 + G50 + B50	Low BL R50 + G50 + B20	Control R50 + G50 + B50	High BL R50 + G50 + B80
2.94	000590-86-3	3-Methyl-butanal	0.045 ± 0.019	0.056 ± 0.02	0.069 ± 0.014	0.039 ± 0.013	0.05 ± 0.022	0.053 ± 0.008
3.08	000096-17-3	2-Methyl-butanal	0.056 ± 0.03	0.076 ± 0.025	0.083 ± 0.22	0.052 ± 0.022	0.07 ± 0.032	0.07 ± 0.012
14.24	000111-84-2	Nonane	1.433 ± 0.95	2.761 ± 2.761	1.209 ± 0.682	1.644 ± 1.271	0.994 ± 0.377	0.848 ± 0.177
24.90	000124-18-5	Decane	0.099 ± 0.067	0.192 ± 0.047	0.192 ± 0.047	0.177 ± 0.168	0.094 ± 0.042	0.129 ± 0.046
25.22	000124-13-0	Octanal	0.291 ± 0.135	0.515 ± 0.178	0.612 ± 0.396	0.318 ± 0.139	0.386 ± 0.1	0.571 ± 0.263
29.67	000122-78-1	Benzeneacet-aldehyde	0.097 ± 0.076	0.068 ± 0.077	0.005 ± 0.004	0.093 ± 0.063	0.204 ± 0.145	0.15 ± 0.146
34.09	000104-87-0	4-Methyl-benzaldehyde	0.109 ± 0.039	0.349 ± 0.259	0.319 ± 0.057	0.481 ± 0.414	0.402 ± 0.255	0.788 ± 0.216
38.13	000124-19-6	Nonanal	0.271 ± 0.254	0.563 ± 0.125	0.5 ± 0.105	0.440 ± 0.119	0.415 ± 0.164	0.706 ± 0.340
45.36	000541-02-6	Decamethyl-cyclopentasiloxane*	0.764 ± 0.229	0.624 ± 0.194	0.53 ± 0.103	0.413 ± 0.162	0.217 ± 0.036	0.269 ± 0.089
47.91	000112-40-3	Dodecane***	0.293 ± 0.021	0.513 ± 0.128	0.43 ± 0.043	0.413 ± 0.154	0.267 ± 0.057	0.407 ± 1.02
48.46	000112-31-2	Decanal	51.977 ± 11.705	62.859 ± 7.064	54.453 ± 12.091	46.08 ± 17.187	47.089 ± 7.865	50.579 ± 14.367
52.34	003913-81-3	(E)-2-decenal	38.3 ± 21.392	27.4 ± 6.057	35.217 ± 19.833	43.078 ± 26.032	46.953 ± 25.316	43.133 ± 10.054
53.10	000112-30-1	1-Decanol	4.521 ± 4.653	2.514 ± 2.138	4.658 ± 3.178	5.201 ± 2.690	1.647 ± 2.704	0.962 ± 0.766
55.64	000112-44-7	Undecanal	0.983 ± 0.366	0.867 ± 0.129	1.007 ± 0.318	1.019 ± 0.730	0.915 ± 0.189	1.058 ± 0.389
58.19	000540-97-6	Dodecamethyl-cyclohexasiloxane*	0.762 ± 0.321	0.643 ± 0.209	0.714 ± 0.067	0.551 ± 0.168	0.296 ± 0.089	0.276 ± 0.088

All extractions were performed for 160 min, and relative percentage of identified compound accumulations expressed as relative percentage (%).

Compounds are listed in order of their elution from a HP-5MS column; RT (retention time in min): on a HP-5MS column. Means (n = 5) followed by the same superscripts in a row of same red light treatments (left part of the table, R20, R50, and R80) or blue light treatments (right part of the table, B20, B50, and B80) indicate significant differences (*p < 0.05 or ***p < 0.001) among control (R50 + G50 + B50) and light treatments.

## Discussion

Yorio et al.^21^ reported that lettuce plants grown under RL supplemented with BL allowed the plants accumulate higher biomass than grown under RL alone. The larger LA could increase the light interception, which contributed to the significant increase in biomass. Kim et al.^22^ demonstrated that BL:RL:GL (4:1:1) increased lettuce LA, which is a good parameter of higher photosynthetic surface area per unit investment in leaf tissue. Although both RL and BL could promote stem elongation^23^, Kong et al.^24^ revealed that BL was more effective than RL in suppressing shoot/leaf elongation in a range of plant species. When grown arugula (*Brassica eruca*) and mustard (*Brassica juncea*) plants under continuous BL (24-h light/0-h dark; PPFD from 20 to 650 μmol m^−2^ s^−1^), the lightings could promote hypocotyl and petiole elongation compared to continuous RL^25^. Wang et al.^26^ also found that when the lettuce photosynthetic performance and growth by stimulating morphological and physiological responses were promoted when plants exposed to BL (200 μmol m^−2^ s^−1^ irradiance). Hernández and Kubota^27^ reported that plant height, hypocotyl length, and epicotyl length of cucumber (*Cucumis sativus*) seedlings were reduced when they were exposed to an increased BL, except for the controls (0% and 100% BL treatments). When the spectral light changes, the morphogenetic and photosynthetic responses are vary among different plant species. A feasibility of tailoring light spectra enables recent plant cultivation technologies and controls the plant growth, development, and nutritional quality. In our study, the growth and morphological features of control-treated coriander plants exhibited a tall appearance, large leaves, and shoot FW, indicating good plant growth under control conditions. Therefore, growth and development in the coriander plant are dependent on the spectral quality of light, and the addition of RL or BL under high (200 μmol m^−2^ s^−1^) and no (0 μmol m^−2^ s^−1^) light intensity may have further decreased plant growth and development due to high light conditions. Perhaps the control conditions achieved a balanced spectral environment for plants without any supplemention of RL or BL at high or low light intensity. Light treatments affected biomass accumulation in the shoot of the coriander plant, and shoot FW accumulation was about 2.5–3.4 times higher for coriander plants under control, high RL, and high BL at high PPFD treatments, than for plants under no RL and no BL treatments at low PPFD. Coriander shoot biomass was reduced under low-PPFD treatments (Fig. 1C, F), suggesting that lowering PPFD might inhibit photosynthesis and carbohydrate production, thus reducing shoot biomass accumulation. Perhaps, plant height, LA, and shoot FW accumulation responses under different combinations of RL and BL lighting may vary among species of crops. Reductions in total coriander FW and LA suggest that light quality and intensity can alter growth, decrease mean weight, and lower market value. Plants raised under the control conditions appeared similar to high market value plants grown in greenhouses, whereas either extra RL or BL-treated plants under high or low light intensity can cause reductions in shoot fresh weight and/or plant size, lowering market value.

Light is an important environmental signal, inducing biosynthesis of photosynthetic pigments and phytochemicals. In this study, the peak emissions of RL, BL, and GL LED closely coincide with the absorption peaks of Chl a and b, and reported wavelengths are at their respective maximum photosynthetic efficiency. Chl has high light absorption at 400–500 and 630–680 nm, and Car has high light absorption at 400–500. Both Chl and Car have low light absorption at 530–610 nm. Hoffmann et al.^28^ demonstrated that BL induces the synthesis of Chl and Car, although Chen et al.^20^ reported that Car levels were not responsive to light quality. Tanaka et al.^29^ found that RL promoted *Cymbidium* leaf growth, but reduced Chl content. In the cucumber, a BL:RL ratio of 1:1 allows optimum leaf development, maximum photosynthesis, and Chl content^29,30^. However, a 1:1 BL:RL ratio did not result in optimal basil growth when compared to any combination of the BL, RL, and GL wavelengths^31^. In our study, Chl and phytochemical contents were reduced as RL or BL lighting was enhanced. It is possible that 200 μmol m^−2^ s^−1^ of RL or BL suppressed Chl, Car, and Ant synthesis in the coriander leaves. It would be interesting to test plant growth, Chl, and phytochemical contents under illumination by various monochromatic light spectra and combinations under a wide range of light intensities. This study provides deeper insight into the interception of light by photosynthetic and photo-protective phytochemicals as a function of light quality and intensity. Antenna complexes include Chl a, Chl b, and Car, and the pigment aggregate acts as an antenna, harvesting the energy of light quanta and delivering this energy to the reaction center^32^. Chls, being non-covalently bound to specific apoproteins, are the major chromophores of most light-harvesting complexes (LHCs), and Chls a and b serve as the major antenna pigments in the LHCs^33^. Caliandro et al.^34^ reported that under photo-oxidative stress, α- and β-branch Car pathways of lutein deficient mutants were detected in whole-plant acclimation for leaf photoprotection. In our study, high BL treatment promoted the contents of Chl a, Chl b, and Car in coriander plants, whereas high RL treatment only increased Chl a and Chl b contents of coriander plants. Notably, lower Car levels of coriander plants were detected in both high BL and high RL treatments compared to no BL and RL treatments and control, suggesting that high BL and RL induce more Chl a and Chl b to absorb the light energy used by coriander plants, thus plants no longer to synthesize Car to help absorb light energy antenna. In addition, higher antioxidant capacity of coriander plants was also observed in high BL treatment. Nevertheless, under no BL and no RL treatments, Car would be synthesized to help absorb light energy antenna.

Johkan et al.^35^ reported that addition of BL in combination with RL induces the accumulation of antioxidative compounds in lettuce plants. Moreover, Son and Oh^36^ found that the ratio of 47% BL + 53% RL LED under 171 ± 7 μmol m^−2^ s^−1^ was important for the morphology, growth, and antioxidative phenolic compounds in lettuce. Wu et al.^37^ discovered that RL LED enhanced the accumulation of antioxidants in pea (*Pisum sativum* L.) seedlings. Similar results were reported by Bliznikas et al.^38^ and they revealed that supplemental RL increased antioxidant levels in parsley and dill plants. De Souza et al. ^39^ suggested that menthol mint (*Mentha arvensis* L.) plants grown under 137 μmol m^−2^ s^−1^ PPFD had the lowest biomass, but produced an essential oil with high level of neomenthol, menthol, and methyl acetate, whereas plants under 543 μmol m^−2^ s^−1^ PPFD had a high biomass and essential oil content, but lower levels of menthol. Ghasemzadeh et al.^40^ stated that total phenolic content of ginger leaves was the highest when plants were grown under 790 μmol m^−2^ s^−1^. Though light treatments were very different, there were some tendencies that enabled an assessment of the underlying mechanisms of the effects observed. For example, Fla, Pha, DPPH scavenging effect, and reducing power increased with increases in PPFD under high RL or BL; thus Fla and Pha contents may be responsible for the antioxidant activity in coriander plants. The intensity of RL or BL increased from 0, 50, to 200 μmol m^−2^ s^−1^ PPFD, and therefore any trend might be a response to RL or BL. Coriander plants had remarkably smaller plant height, LA, and Chl, Car, and Ant contents when treated with high RL or BL intensity. However, coriander plants grown under control conditions were taller, had larger leaves, and contained higher shoot FW, Chl, Car, and Ant contents. Different LED spectra are capable of triggering various morphological, phytochemical reactions, and antioxidant properties. Decreases in plant height and LA in coriander plants under higher RL or BL levels were the result of lowered Chl (Fig. 2), Car, and Ant contents (Fig. 3), but greater Fla and Pha levels (Fig. 4B–D), DPPH scavenging, and reducing power (Fig. 5A–D) when these plants were subjected to high PPFD. These results suggest that stronger BL intensity might better penetrate the plant canopy than weaker BL intensity, rendering higher levels of Fla, Pha, DPPH scavenging effect, and reducing power. Blue light scattering is stronger than red light by carries a higher energy than red light and therefore carries a greater risk of the over-excitation. Coriander leaves appear to be sensitive to higher RL or BL intensity, which caused serious photoinhibition and photodamage, avoidance of excessive energy absorption in response to higher photosynthesis photochemical efficiencies, ultimately leading to lower plant height and LA in comparison to controls. However, the response of higher Fla, Pha, DPPH scavenging, and reducing power values was related to lower Chl and Ant contents of coriander plants under higher RL or BL intensity treatment. Protective mechanisms in coriander plants should prevent its leaves from excessive reduction in PSII acceptors. The assessment of Chl, Car, and Ant parameters under varying light quality and intensity is an important tool for understanding how to improve the photosynthetic productivity of plants. The application of light quality and intensity could beneficially influence growth, Chl, Car, and Ant contents in mature coriander plants, and properly selected RL plus BL LED could be used to produce antioxidant-rich spice culture.

Controlling light quality and intensity is effective in optimizing the yield and quality of coriander plants. Currently, there is little information available on the effect of light treatments on the accumulation of flavor compounds in coriander. Variations of the essential oil constituents in *C. sativum* have been observed, depending on genetic and environmental factors as well as tissue part, ontogeny, and analytical methods^41^. Several studies have shown that the major identified volatile compounds in essential oil of coriander leaves were 2-dodecenal, decanal, E-2-decenal, linalool, tetradecenal, 2-decen-1-ol, dodecan-1-ol, undecenal, dodecane, E-2-tridecenal, trans-tetradec-2-enal, E-2-hexadecenal, cyclodecane, menthone, pentadecenal, and pinene^42,43^. Shahwar et al.^44^ identified 27 and 21 compounds from coriander leaves and seeds, respectively. Among them, there were only 10 compounds from coriander leaves identified in our study as shown in Table S2, and the rest of compounds of coriander leaves under the light treatments were unique in our study. Shahwar et al.^44^ reported that decenal is one of the major volatile compounds identified in coriander leaves, and is an essential oil that produces the oily, sweet, or grassy odor of the plant. Liu et al.^45^ also indicated that decenal had antioxidant activity and inhibitory of bactericidal effects on the microorganisms. Sankhuan et al.^46^ statted that *Artemisia annua* plants grown under blue spectra at 445 nm had higher leaf fresh weight, increased amounts of artemisinin, and enhanced production of several terpenoids against the malarial parasite *Plasmodium falciparum* NF54, whereas red spectrum at 660 nm led to decreased production of bioactive compounds and decreasing anti-malarial activity. However, high RL led to significantly diminished productions of decanal compared with other light treatments. The antioxidant activity and the inhibitory of bactericidal function of octanal have been reported by Liu et al.^45^. In our study, plants grown under low BL exhibited significantly decreases in the amounts of decenal, suggesting that coriander plants growth under high RL or low BL condition would decrease the bactericidal compounds. Interestingly, (E)-2-decenal was the second highest compound area (27.4 ± 6.057%, RT 52.34 min) identified in our study, but it was the highest compound area detected in their coriander leaves. Apparently, both decenal and (E)-2-decenal can be useful for the production of essential oil from coriander leaves. Naidu and Priyadarshi^47^ also revealed that the most common compounds identified by GC–MS in two popular varieties of *Coriandrum sativum* were decanal, 2-decenal, undecanal, dodecanal, E-2-dodecenal, E-2-tridecenal, tetradecanal, E-9-tetradecenal, 7-hexadecenal, and n-hexadecanoic acid. The cyclooxygenase-2 (COX-2) is an enzyme responsible for formation of prostanoids, and pharmaceutical inhibition of COX-2 can provide relief from the symptoms of inflammation and pain^48^. Dodecane has been isolated from the essential oils of various plants including ginger (*Zingiber officinale*) with strong inhibitory effects on COX-2 enzyme activity^48^. Our results show that there has been a significant decrease in the amounts of dodecane, the levels of antioxidants, Chl, Car, Ant, and Fla, and the plant heights as well when the plants grown under high RL conditions, implying that antibacterial effectiveness and economic efficiency of the metabolites production may not be maximized by the management of light treatment under high RL conditions.

In our study, among 15 identified compounds (Table 1), the highest compound of DCP, DO, and DOCP was detected in low RL, control, and low BL treatments, respectively, suggesting that coriander plants responding to light treatment with a low intensity of RL or BL exhibited an enhanced accumulation of both DCP and DOCP in cells. Since both DCP and DOCP metabolism in coriander leaves are more sensitive to light intensity than DO metabolism, the biosynthesis of DCP and DOCP in coriander leaves can be optimally activated by applying such light stimuli. Thus, an understanding of the effects of the light treatments upon the content of DCP, DO, and DCP compounds in coriander plants is an important aspect of the strategic implementation of their production and culinary and clinical applications. Differing responses in Chl, Car, Ant, antioxidants, and metabolite accumulations for optimizing plant growth and development in a controlled-climate setting are dependent upon various light quality and intensity culture systems, which may be used to satisfy commercial requirements for rapid, large-scale, and precise management of coriander plant production. Various growth-regulating strategies can be carefully chosen to produce the desired features of coriander for use as cooking materials, raw materials, or medicinal materials.

## Conclusions

WE investigated the effective light treatments for growing coriander plants, and found that various light quality and intensity combinations influenced the accumulations of Chl, Car, Ant, Fla, and Pha, and mediated antioxidative properties and secondary metabolites in coriander leaves. In agricultural production, yields and costs are the two most important criteria in efforts to optimize environmental factors. The control resulted in many positive effects on the growth, development, and appearance of coriander plants, and increases of Chl, Car, Ant contents, suggesting that control treatment (R50 + G50 + B50) with 50 μmol m^−2^ s^−1^ PPFD of RL, BL and GL is optimal for the coriander plants. In contrast, high BL (R50 + G50 + B200) with 200 μmol m^−2^ s^−1^ PPFD of BL induced Fla, Pha, DPPH scavenging effect, and reducing power responses. Furthermore, the accumulation of DCP and DOCP was detected in low RL (R20 + G50 + B50) and low BL (R50 + G50 + B20) with 20 μmol m^−2^ s^−1^ PPFD of RL and BL, respectively. These results can be useful in developing a new LED lighting apparatus optimized for coriander production in plant factories, and may also benefit fundamental research, providing a better understanding of the photosynthetic characteristics and commercial applications of food production.

## Materials and methods

### Plant material and cultural practice

The seeds of the coriander plant (*Coriandrum sativum,* cv. RV-164) were purchased from Known-You seed company (Kaoshiung, Taiwan). The coriander seeds were germinated in hydroponic condition with rockwool cubes (2.5 cm × 2.5 cm × 3.0 cm) for 1 week in a 25 °C growth chamber. The seedlings were illuminated with cool white fluorescent lamps (light intensity of 10 μmol m^−2^ s^−1^ PPFD) for 12 h per day. When the plant height reached 1 cm, they were transferred into polystyrene foam cubes, mounted in Styrofoam plates (eight holes per plate) and placed in a container (105 cm × 60 cm × 9 cm). The aerated complete nutrient solution^6^ was supplied continuously in a commercial hydroponic growth room (Nano-Bio Light Technology Co., LTD., New Taipei, Taiwan). The nutrient solution was renewed every week and adjusted to pH 6.5–7.0 and an electrical conductivity of 2–3 mS cm^−1^. All treatments were maintained throughout the experiment at a temperature of 25 °C, a 12 h daily photoperiod, and 75% relative humidity in the growth chamber.

### Light treatments

Light quality treatments for coriander seedling growth in the above mentioned hydroponic system consisted of five following commercially available mixtures of LED arrays designed by Hi-Point Technology (Kaoshiung, Taiwan):

1. GL (50 μmol m^−2^ s^−1^) + BL (50 μmol m^−2^ s^−1^) as no RL treatment (short as G50 + B50),
2. RL (50 μmol m^−2^ s^−1^) + GL (50 μmol m^−2^ s^−1^) + BL (50 μmol m^−2^ s^−1^) as control treatment, (short as R50 + G50 + B50),
3. RL (200 μmol m^−2^ s^−1^) + GL (50 μmol m^−2^ s^−1^) + BL (50 μmol m^−2^ s^−1^) as high RL treatment (short as R200 + G50 + B50),
4. RL (50 μmol m^−2^ s^−1^) + GL (50 μmol m^−2^ s^−1^) as no BL treatment (short as R50 + G50), and
5. RL (50 μmol m^−2^ s^−1^) + GL (50 μmol m^−2^ s^−1^) + BL (200 μmol m^−2^ s^−1^) as high BL treatment (short as R50 + G50 + B200).

Each light quality was run in a growth room under the light intensity of 0, 50 (control), and 200 μmol m^−2^ s^−1^ PPFD which was measured daily above the plant canopy and maintained by adjusting the distance of the LED to the plant canopy. Spectral distributions of BL (with a peak at 460 nm), RL (at 625 nm), and GL (at 530 nm) were measured with a spectral radiometer (Hi-Point Technology, Kaoshiung, Taiwan) in the 300–800-nm range. The lamp’s luminous area was 120 × 60 cm, and the lamp contained BL, RL and GL LED with 30 pieces in each LED color, and the spectrum was recorded at the top of the coriander packs. All treatments were maintained at a constant 25 °C with a 12/12-h light/dark photoperiod and were procured on January 2020. Plants were harvested 45 days after sowing. Shoot samples were freeze-dried in a vacuum freeze dryer (LabConco, Tokyo, Japan) and ground into a powder with a mortar and pestle before extraction and antioxidant analysis. Twenty coriander plants were randomly planted under each light source with a light intensity, and 5 plants from each light treatment were used for the following growth and physiological parameter measurements.

### Plant growth measurements

Plant height (cm) was measured from the base of the plant to the top of the stem with a Vernier caliper. The shoot fresh weight (FW, g) was measured with an electronic balance. The leaf area (LA, cm^2^) was measured by an LA meter (LI-3100, LI-COR, Lincoln, NE, USA).

### Determination of pigments, phyotochemicals, and antioxidant activities

The concentrations of Chl and Car in coriander leaves were determined as described^20^. In Brief, leaf sample powder (0.01 g) was mixed with 80% acetone (5 mL) and incubated at 4 °C overnight, and was centrifugation at 13,000 g for 5 min. The supernatants were transferred to a new tube, and then used to determine the Chl a and Chl b absorbance in acetone at 663.6 nm and 646.6 nm, respectively, using a spectrophotometer (Hitachi U-2000, Tokyo, Japan). Following equations were used to calculate the Chl a, Chl b, Chl a + b, and Car concentrations:

$$ {\text{Chl a \,concentration}} = \, \left( {{12}.{\text{25 A}}_{{{663}.{6}}} - { 2}.{\text{55 A}}_{{{646}.{6}}} } \right)\; \times \;{\text{volume of supernatant }}\left( {{\text{mL}}} \right)/{\text{sample weight }}\left( {\text{g}} \right) $$

$$ {\text{Chl b \,concentration}} = \, \left( {{2}0.{\text{31 A}}_{{{646}.{6}}} - { 4}.{\text{91 A}}_{{{663}.{6}}} } \right)\; \times \;{\text{volume of supernatant }}\left( {{\text{mL}}} \right)/{\text{sample weight }}\left( {\text{g}} \right) $$

$$ {\text{Chl a}} + {\text{b \,concentration}} = \, \left( {{17}.{\text{76 A}}_{{{646}.{6}}} + { 7}.{\text{34 A}}_{{{663}.{6}}} } \right)\; \times \;{\text{volume of supernatant }}\left( {{\text{mL}}} \right)/{\text{sample weight }}\left( {\text{g}} \right) $$

$$ {\text{Car \,concentration}} = [\left( {{4}.{\text{69 A}}_{{{44}0.{5}}} } \right)\; \times \;{\text{volume of supernatant }}\left( {{\text{mL}}} \right)/{\text{sample weight }}\left( {\text{g}} \right)]{-}0.{267 }\left( {{\text{Chl a}} + {\text{b}}} \right) $$

The anthocyanin (Ant) concentrations of extracts were measured as described by Chen et al.^20^. The extraction buffer (99% methanol containing 1% HCl) was added to leaf sample powder and incubated at room temperature for 1 h. The mixture was then centrifuged at 3000 rpm for 5 min (4 °C), and the supernatant was transferred to a new tube, followed by measuring absorbances at 530 nm and 657 nm on a spectrophotometer (Hitachi U-2000, Tokyo, Japan). Following equation was used to calculate the Ant concentration: Ant concentration (μmol g^−1^ DW) = (A_530_−0.33 × A_657_/31.6) × volume of supernatant (mL)/sample weight (g).

The total phenolic acid (Pha) concentrations of extracts were determined as described by Bantis et al.^14^. In short, gallic acid standard (Sigma, St. Louis, MO, USA) and an aliquot of the acidic methanolic extract were diluted with acidified methanol solution containing 1% HCl. The sample extracts (100 μl) were mixed with 2% Na_2_CO_3_ (2 mL) and allowed to equilibrate for 2 min before adding 50% Folin-Ciocalteu reagent, and measured the absorbance at 750 nm using the Varioskan Flash Multimode Reader (Thermo Scientific, Rockford, IL, USA).

Total flavonoids (Fla) in extracts were determined by the method of Lin et al.^6^. One mL of methanol extract (0.5 mg mL^−1^) was mixed with 1 mL aluminum chloride (2%). The mixture was mixed gently at room temperature for 15 min, and the absorbance at 540 nm was collected with a spectrophotometer (Hitachi U-2000, Tokyo, Japan).

The free 2,2-Diphenyl-1-picrylhydrazyl (DPPH) radical-scavenging ability was measured using protocols described by Tang et al. ^49^. Briefly, the powder of lyophilized coriander leaf sample (0.01 g) was extracted with of 40% ethanol (5 mL) at room temperature, and then filtered with Whatman filter paper (No. 1). The remaining residue was re-extracted three times with 40% ethanol until the residue was colorless. An aliquot of the ethanolic extract (0.05 mL) with a serial dilution was added to a 0.4 mM DPPH solution (0.15 mL). The mixture was mixed and kept in dark for 90 min. The color of mixture was changed from purple (DPPH radical) to yellow (DPPH-H) after reduction, which can be quantified by a decrease in absorbance at 517 nm. Following equation was used to calculate the radical-scavenging activity of total free radicals: Scavenging activity (%) = [(absorbance of a blank DPPH solution−absorbance of a sample)/(absorbance of a blank DPPH solution)] × 100. The blank was conducted in the same manner, except that ethanol was used instead of a sample, and 40% ethanol was used as the blank. The scavenging effect was obtained by plotting the percentage of residual DPPH at steady state as a function of the antioxidant concentration.

The reducing properties of a serial dilution of the extract were determined using protocols described by Huang et al.^19^. In brief, the ethanolic extract (0.05 mL) was mixed with 0.2 M sodium phosphate buffer pH = 6.6 (0.05 mL) and 1% potassium ferrocyanide (0.033 mL), and incubated at 50 °C for 20 min. Then, 10% trichloroacetic acid (0.05 mL) was added to the mixture, and centrifuged at 1500 g for 10 min. Supernatant (0.1 mL) was transferred to a new tube and added distilled-deionized water (0.1 mL) and 0.1% ferric chloride (0.02 mL). After 10 min, the absorbance at 700 nm was measured against a blank. Following equation was used to calculate the reduction capacity:

Reduction capacity = sample of A700−blank of A700.

### Analysis of secondary metabolite compounds of coriander leaves

This experiment was conducted in the same culture practice as described above in “Plant material and cultural practice”, and was procured on November 2020. However, light quality and intensity treatments were adjusted to lower than the above-mentioned light treatments (“Light treatments”) for improved coriander plant growth and development, because we observed that high RL and BL intensity treatments at 200 μmol m^−2^ s^−1^ and no RL and BL intensity treatments had adversely affect coriander growth and development. Thus, the light treatments in this experiment were following:

1. RL (50 μmol m^−2^ s^−1^) + GL (50 μmol m^−2^ s^−1^) + BL (50 μmol m^−2^ s^−1^) as control treatment (short as R50 + G50 + B50),
2. RL (20 μmol m^−2^ s^−1^) + GL (50 μmol m^−2^ s^−1^) + BL (50 μmol m^−2^ s^−1^) as low RL treatment (short as R20 + G50 + B50),
3. RL (80 μmol m^−2^ s^−1^) + GL (50 μmol m^−2^ s^−1^) + BL (50 μmol m^−2^ s^−1^) as high RL treatment (short as R80 + G50 + B50),
4. RL (50 μmol m^−2^ s^−1^) + GL (50 μmol m^−2^ s^−1^) + BL (20 μmol m^−2^ s^−1^) as low BL treatment (short as R50 + G50 + B20), and
5. RL (50 μmol m^−2^ s^−1^) + GL (50 μmol m^−2^ s^−1^) + BL (80 μmol m^−2^ s^−1^) as high BL treatment (short as R50 + G50 + B80).

Plants were harvested 45 d after sowing. Five replicates of plants were randomly selected from each light treatment and used for the chemical composition of coriander leaves.

Leaf samples were freeze-dried and ground into fine powder by mortar and pestle. The grounded leaf powder (0.1 g) was then transferred to a 10 mL glass vial, sealed, and heated to 60 °C. The extraction of volatile compounds was performed by a commercial SPME (Solid Phase Micro-Extraction) device (Supelco, Bellefonte, PA, USA), using fibers of 50/30 μm thickness, divinylbenzene/carboxen/polydimethylsiloxane (DVB/CAR/PDMS). Before the SPME fiber was inserted into the vial, the sample was equilibrated for 15 min at the extraction temperature of 60 °C. Prior to analysis, the SPME fiber was preconditioned in the injection port of the gas chromatograph at the 250 °C. This temperature was the same as that set for the injection port of the gas chromatograph during desorption. Analyses were performed using Agilent 6890/Agilent 5975B (Agilent, Paolo Alto, CA, USA) gas chromatography/mass spectrometric (GC-MS) analyses, fused HP-5MS column (30 m × 0.25 mm; 0.25 μm film thickness). Helium was used as carrier gas at a flow rate of 1 mL min^−1^. The MS operating parameters consisted of an ionization voltage of 70 eV and an ion source temperature of 230 °C. The column temperature was 35 °C (5 min) to 180 °C (10 min) at the rate of 1°C min^−1^. Compound identification was accomplished by comparing the National Institute of Standards and Technology (NIST) library data of the peaks with those reported in the literature. All extractions were performed for 160 min, and peak areas of identified compound accumulation were expressed in area (absorbance). All compounds were identified by comparison of their chromatographic parameters and mass spectra with authentic standards.

### Statistical analysis

The collected measurements including morphological and physiological parameters, antioxidant capacity, and chemical composition were analyzed by a completely randomized analysis of variance (ANOVA). The PC SAS ver. 9 (SAS Institute, Cary, NC, USA) was used to analyzed the collected measurements here. The means were separated by the least significant difference (LSD) test at *p* ≤ 0*.*05 and the results were expressed as the mean ± standard deviation (SD).

### Ethical approval

We confirm that all methods were performed in accordance with the relevant guidelines and regulations by including a statement in the Methods section to this effect. Experimental research on plants, including the collection of plant material, agrees with relevant institutional, national, and international guidelines and legislation.

## Supplementary Information

Supplementary Tables.

## Data Availability

The authors confirm that the data supporting the findings of this study are available within the article and/or its supplementary materials.
