# Supplementary material for: Physiological responses and antioxidant properties of coriander plants (Coriandrum sativum L.) under different light intensities of red and blue lights
Source: Sci Rep. 2022 Dec 7;12:21139. doi: 10.1038/s41598-022-25749-3 (PMC9729621; doi:10.1038/s41598-022-25749-3)
Supplement: Supplementary file 1 — Supplementary Tables. [file 41598_2022_25749_MOESM1_ESM.doc]

**Supplementary Table S1** The chemical composition of coriander leaf essential oil extracted from freeze-dried samples of 5 plants under different light treatments. All extractions were performed for 160 min, and peaks of identified compounds were presented

| RT (min) | CAS number | Compound | low RL  R20+G50+B50 | control R50+G50+B50 | high RL R80+G50+B50 |  | low BL  R50+G50+B20 | control R50+G50+B50 | high BL R50+G50+B80 |
| --- | --- | --- | --- | --- | --- | --- | --- | --- | --- |
| 7.204 | 000111-65-9 | Octane | ○ | ○ | ○ |  | ○ | ○ | ○ |
| 7.283 | 000066-25-1 | Hexanal | ○ | ○ | ○ |  | ○ | ○ | ○ |
| 8.405 | 002213-23-2 | 2,4-dimethyl-heptane | ○ | ○ | ○ |  | ○ | ○ | ○ |
| 10.465 | 006728-26-3 | (E)-2-hexenal | ○ | ○ | ○ |  | ○ | ○ | ○ |
| 14.433 | 000111-71-7 | Heptanal | ○ | ○ | ○ |  | ○ | ○ | ○ |
| 26.253 | 000556-67-2 | Octamethyl-cyclotetrasiloxane | ○ | ○ | ○ |  | ○ | ○ | ○ |
| 29.923 | 000122-78-1 | Benzeneacetaldehyde | ○ | ○ | ○ |  | ○ | ○ | ○ |
| 31.579 | 000099-85-4 | γ-terpinene | ○ | ○ | ○ |  | ○ | ○ | ○ |
| 34.085 | 000104-87-0 | 4-methyl-benzaldehyde | ○ | ○ | ○ |  | ○ | ○ | ○ |
| 48.715 | 000112-31-2 | Decanal | ○ | ○ | ○ |  | ○ | ○ | ○ |
| 48.946 | 000432-25-7 | β-cyclocitral | - | - | ○ |  | - | - | ○ |
| 51.41 | 001014-60-4 | 1,3-di-tert-butylbenzene | ○ | ○ | ○ |  | ○ | ○ | ○ |
| 54.56 | 004179-38-8 | 2-n-octylfuran | ○ | ○ | ○ |  | ○ | ○ | ○ |
| 64.19 | 000112-54-9 | Dodecanal | ○ | ○ | ○ |  | ○ | ○ | ○ |
| 83.685 | 000124-25-4 | Tetradecanal | ○ | ○ | ○ |  | ○ | ○ | ○ |

The symbol of ○ indicates that the identified compound was detected only one or two times from freeze-dried samples of 5 plants, therefore, it is not possible to calculate the significant differences between light treatments. Moreover, the symbol of - indicates that the compound was not identified from any of the samples (n = 5). Compounds are listed in order of their elution from a HP-5MS column; RT (retention time in min): on a HP-5MS column.

**Supplementary Table S2** Comparison of the identified compounds of coriander between Shahwar et al. (2012) (shown as Area %) and our study (shown as CAS number and RT). Twenty-seven and 21 compounds were identified from coriander leaves and seeds, respectively, by Shahwar et al. (2012) [41]

| Compound identified^1^ | Area (%)^1^ | CAS number^3^ | RT (min)^3^ |  | Compound identified^2^ | Area (%)^2^ | CAS number^3^ | RT (min)^3^ |
| --- | --- | --- | --- | --- | --- | --- | --- | --- |
| (E)-2-Decenal | 32.23 | 003913-81-3 | 52.34 |  | Linalool | 55.49 |  |  |
| Linalool | 13.97 |  |  |  | γ-Terpinene | 7.47 | 000099-85-4 | 31.579 |
| (E)-2-Dodecenal | 7.51 |  |  |  | α-Pinene | 7.14 |  |  |
| (E)-2-Tetradecenal | 6.56 |  |  |  | Camphor | 5.59 |  |  |
| 2-Decen-l-ol | 5.45 |  |  |  | Decanal | 4.69 | 000112-31-2 | 48.46 |
| (E)-2-Undecenal | 4.31 |  |  |  | Geranyl acetate | 4.24 |  |  |
| Dodecanal | 4.07 | 000112-54-9 | 64.19 |  | Limonene | 3.1 |  |  |
| (E)-2-Tridecenal | 3.00 |  |  |  | Geraniol | 2.23 |  |  |
| (E)-2-Hexadecenal | 2.94 |  |  |  | Camphene | 1.78 |  |  |
| Pentadecenal | 2.47 |  |  |  | D-Limonene | 1.36 |  |  |
| Undecanal | 2.43 | 000112-44-7 | 55.64 |  | Myrcene | 0.98 |  |  |
| 1-Decanol | 2.18 | 000112-30-1 | 53.1 |  | ρ-Cymene | 0.9 |  |  |
| α-Pinene | 1.90 |  |  |  | Decanol | 0.81 |  |  |
| Decanal | 1.73 | 000112-31-2 | 48.46 |  | α-Terpinol | 0.81 |  |  |
| Carvone | 1.40 |  |  |  | (E)-2-Dodecenal | 0.79 | 003913-81-3 | 52.34 |
| 1-Eicosanol | 1.35 |  |  |  | β-Pinene | 0.71 |  |  |
| Tridecanal | 1.09 |  |  |  | 2-Decenal | 0.63 |  |  |
| Tetradecanal | 1.01 | 000124-25-4 | 83.685 |  | Bornel | 0.41 |  |  |
| 2-Undecen-l-ol | 0.83 |  |  |  | Sabinene | 0.4 |  |  |
| 4-Dodecenal | 0.74 |  |  |  | α-Thujene | 0.28 |  |  |
| Hexadecenal | 0.70 |  |  |  | 1, 8 Cineol | 0.19 |  |  |
| Nonane | 0.51 | 000111-84-2 | 14.24 |  |  |  |  |  |
| 1-Tetracosanol | 0.48 |  |  |  |  |  |  |  |
| Germacrene | 0.35 |  |  |  |  |  |  |  |
| Diallyfumerate | 0.34 |  |  |  |  |  |  |  |
| Heptadecenal | 0.30 |  |  |  |  |  |  |  |
| Phytol methyl stearate | 0.15 |  |  |  |  |  |  |  |

^1^ Compound identified and Area (%) of coriander leaves are modified from Table 2 of Shahwar et al. (2012), and ranked according to the Area (%).

^2^ Compound identified and Area (%) of coriander seeds are modified from Table 3 of Shahwar et al. (2012), and ranked according to the Area (%).

^3^ The CAS number and RT (min) of coriander leaves indicate 10 compounds identified in our study.
